# Supplementary material for: The interactome of intact mitochondria by cross-linking mass spectrometry provides evidence for coexisting respiratory supercomplexes
Source: Mol Cell Proteomics. 2017 Dec 8;17(2):216–32. doi: 10.1074/mcp.RA117.000470 (PMC5795388; doi:10.1074/mcp.RA117.000470)
Supplement: Supplemental Data [file supp_RA117.000470_133922_0_supp_23978_fzffwf.zip › spectra_annotation/mito_DR_spectra_annotation/465-1-7-1-94-1.pdf]

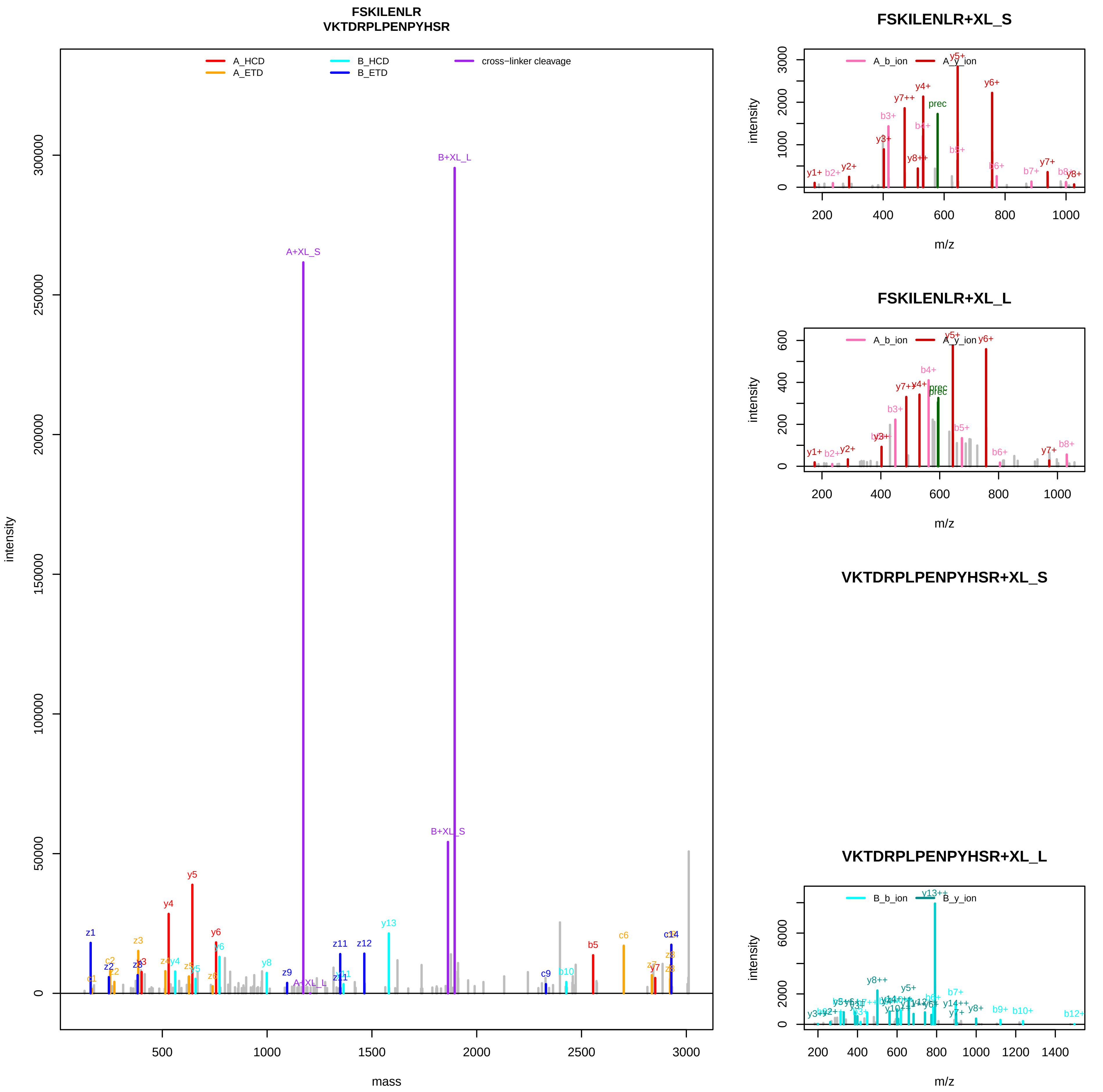

FSKILENLR
VKTDRPLPENPYHSR
A_HCD
A_ETD
B_HCD
B_ETD
cross−linker cleavage
intensity
mass
0
50000
100000
150000
200000
250000
300000
500
1000
1500
2000
2500
3000
A+XL_S
B+XL_L
B+XL_S
A+XL_L
y4
y5
y6
y8
y13
z1
z2
z3
z9
z11
z12
c9
b10
b5
c6
c14
y7
FSKILENLR+XL_S
FSKILENLR+XL_L
VKTDRPLPENPYHSR+XL_S
VKTDRPLPENPYHSR+XL_L
A_b_ion
A_y_ion
B_b_ion
B_y_ion
prec
m/z
intensity
y5+
y6+
y4+
y7++
b3+
b4+
y3+
y8++
b5+
b6+
b7+
b8+
y7+
y8+
y1+
b2+
y2+
y13++
y8++
b7+
y14++
y8+
b9+
b10+
b12+
